# Bioactive Compounds, Antioxidants, and Health Benefits of Sweet Potato Leaves

**DOI:** 10.3390/molecules26071820

**Published:** 2021-03-24

**Authors:** Hoang Chinh Nguyen, Chang-Chang Chen, Kuan-Hung Lin, Pi-Yu Chao, Hsin-Hung Lin, Meng-Yuan Huang

**Affiliations:** 1 Faculty of Applied Sciences, Ton Duc Thang University, Ho Chi Minh City 700000, Vietnam; nguyenhoangchinh@tdtu.edu.vn; 2 National Research Institute of Chinese Medicine, Ministry of Health and Welfare, Taipei 11221, Taiwan; chencc@nricm.edu.tw; 3 Department of Horticulture and Biotechnology, Chinese Culture University, Taipei 11114, Taiwan; rlin@ulive.pccu.edu.tw; 4 Department of Nutrition and Health Sciences, Chinese Culture University, Taipei 11114, Taiwan; pychao@ulive.pccu.edu.tw; 5 Department of Life Sciences, National Chung Hsing University, Taichung 40227, Taiwan

**Keywords:** antioxidants, bioactive compounds, health benefits, phytochemicals

## Abstract

Sweet potato (*Ipomoea batatas*) is one of the most important food crops worldwide and its leaves provide a dietary source of nutrients and various bioactive compounds. These constituents of sweet potato leaves (SPL) vary among varieties and play important roles in treating and preventing various diseases. Recently, more attentions in health-promoting benefits have led to several in vitro and in vivo investigations, as well as the identification and quantification of bioactive compounds in SPL. Among them, many new compounds have been reported as the first identified compounds from SPL with their dominant bioactivities. This review summarizes the current knowledge of the bioactive compositions of SPL and their health benefits. Since SPL serve as a potential source of micronutrients and functional compounds, they can be further developed as a sustainable crop for food and medicinal industries.

## 1. Introduction

Sweet potato (*Ipomoea batatas* [L.] Lam.) is considered to be a major food crop worldwide, and it is widely produced and consumed in East Asia, Oceania, and Sub-Saharan Africa, with the highest production in China which comprises 76.07% of the world’s production [1,2]. Sweet potato leaves (SPL) are considered to be a leafy vegetable consumed by humans, which is currently widely used for food due to its high yield, drought tolerance, and the ability to grow in different climates and farming systems [3,4]. SPL contain essential minerals of Na, Mg, P, Ca, and K with ranges of 8.06−832.31, 220.2−910.5, 131.1−2639.8, 229.7−1958.1, and 479.3−4280.6 mg/100 g DW, respectively, while the minerals Cu, Zn, Mn, and Fe have ranges of 0.7−1.9, 1.2−3.2, 1.7−10.9, and 1.9−21.8 mg/100 g DW, respectively (Table 1) [5]. Leaves of sweet potato hold niacin (856−1498 μg/100 g), vitamins B6 (120−329 μg/100 g), B2 (248−254 μg/100 g), B1 (53−128 μg/100 g), C (62.7−81 mg/100 g), E (1.39−2.84 mg/100 g), pantothenic acid (320−660 μg/100 g), β-carotene (273−400 μg/100 g), and biotin (3−8 μg/100 g), having higher contents of vitamins B2, C, E, biotin, and β-carotene than the plant’s stems and stalks [2].

In addition to their nutritional values, SPL have been found as a functional food containing various bioactive compounds that provide a variety of health-promoting benefits [6]. Many studies on functional compounds of SPL indicate that their health benefits are related to high levels of polyphenols, flavonoids, and carotenoids [7,8,9]. These compounds exhibit various bioactivities, such as antioxidant [10,11,12,13,14], anti-cancer [15,16,17], anti-mutagenic activities [18], immune modulation [19], and hepato-protection [20] (Table 2). A full understanding of the bioactive compositions and health benefits of SPL can provide improvements in sweet potato utilization and commercialization. Thus, the growing interest in the identification of phytochemicals in SPL and their influence on human health have recently resulted in a large number investigations, many of which are summarized in this review.

## 2. Bioactive Compounds in Sweet Potato Leaves

SPL are recognized as one of the most important sources of polyphenols [21,22] with various constituents [23,24,25]. Among them, caffeic acid and caffeoylquinic acid derivatives, such as 4,5-di-O-caffeoylquinic acid, 3,4,5-tri-O-caffeoylquinic acid, 3-mono-O-caffeoylquinic acid, 3,4-di-O-caffeoylquinic acid, and 3,5-di-O-caffeoylquinic acid, are indicated as the main phenolic constituents in SPL [9,26,27]. These constituents are associated with the specific genotypes and the stages of leaf development [9,23,26,28]. The level of polyphenols in SPL varies among their varieties ranging from 0.3−13.5 g gallic acid equivalent (GAE)/100 g DW, which is 7–9 times higher than that levels found in grape seeds [29]. The leaf total phenolic compounds (TPC) of eight sweet potato varieties from Japan ranged from 6.3–13.5 g GAE/100 g DW [10], whereas the TPC in leaves of four Taiwanese varieties and/or cultivars (‘TNG10’, ‘TNG57’, ‘TNG66’, and ‘YSP’) is relatively low [30]. The TPC in SPL cultivated in China and Portugal was 0.9–2.7 g chlorogenic acid equivalent (CAE)/100 g DW [17] and 1.20−1.32 g GAE/100 g DW [31], respectively.

The phenolic and flavonoid contents of SPL are affected by the level of light exposure. The content of hydroxybenzoic acids (p-anisic acids and benzoic), hydroxycinnamic acids (sinapic acid and p-coumaric acid conjugates), anthocyanins, catechins, and flavonols in SPL are dramatically increased under a long day photoperiod [72,73].

SPL are harvested several times during the growth of sweet potato, and the phenolic composition and antioxidant activities of SPL may vary among the stages of SPL development. Therefore, selection a suitable period for harvesting SPL is important to obtain SPL with high level of phenolic constituents and antioxidant activities. Suárez et al. [74] compared the nutritional and phenolic compositions of SPL harvested in three different periods—August 21 (T1), September 6 (T2), and September 21 (T3)—and found that SPL harvested in T3 had the highest TPC (9.1 ± 0.3 g/100 g DW), vitamin E content (5.8 ± 0.4 mg/100 g DW), vitamin C content (104.6 ± 4.9 mg/100 g DW), and the antioxidant activity, compared to those in T1 and T2. Furthermore, among the phenolic compositions, 2 flavonoids (quercetin and isoquercetin), 1 caffeic acid, and 6 caffeoylquinic acids (3,4,5-tri-O-caffeoylquinic acid, 3,4-di-O-caffeoylquinic acid, 3-O-caffeoylquinic acid, 3,5-di-O-caffeoylquinic acid, 4,5-di-O-caffeoylquinic acid, and 5-O-caffeoylquinic acid) were significantly different in those three harvest periods [74].

Similar to other leafy vegetables, fresh SPL are easily decayed after harvesting, which lowers thier nutritional values and bioactive compounds. Therefore, SPL are proposed to be dried to prolong their shelf-life for industrial applications. The effect of drying method (freeze drying and drying at different temperatures) on the content of caffeoylquinic acid derivatives in SPL was investigated by Jeng et al. [11]. The freeze-drying treatment resulted in the highest amount of caffeoylquinic acid derivatives (147.84 mg/g), whereas the caffeoylquinic acid derivatives contents of SPL were significantly reduced by using drying methods at both 70 °C (58.26 mg/g) and 100 °C (20.53 mg/g) [11]. These results imply that drying at low temperatures (<30 °C) may be a suitable method to maintain the nutritional value and bioactive compounds in SPL [15]. In addition, Sui et al. [75] investigated the influences of vacuum-freeze, hot-air, and microwave-vacuum drying methods on the nutritional composition of SPL, and indicated that the vacuum-freeze drying method maintained the highest vitamins (B1, B2, C, and E), minerals (Zn, P, and Mg), total dietary fiber, and TPC.

SPL are commonly used for human diet by dosmestic cooking; therefore, it is crucial to understand the effect of domestic cooking on the the level of polyphenols and antioxidant activity of SPL. Sun et al. [36] studied the influences of different cooking methods, including baking, steaming, boiling, frying, and microwaving, on individual phenolic compound, TPC, and antioxidant activity of SPL. Among these tested cooking methods, steaming showed the highest TPC, whereas boiling resulted in the lowest TPC in SPL, indicating that steaming is the most efficient cooking method to maintain levels of polyphenols and antioxidant activity in SPL [36].

Extraction is an initial step in the separation and purification process to obtain bioactive compounds from biomass materials for further applications. To maximize the level of a target component and biological activities, the most suitable solvent should be selected for the extraction. Fu et al. [21] investigated the influence of ten different solvents (water, aqueous ethanol, aqueous methanol, and aqueous acetone) on the recovery of polyphenols from SPL, and showed that SPL extract produced by using 50% acetone resulted in the highest TPC (43.8 mg CAE/g DW) and the strongest antioxidant activities, whereas SPL extract using 70% ethanol contained the highest total flavonoid (3.4 mg quercetin equivalents (QE)/g DW) and total anthocyanin content (36.5 mgcyanidin-3-glucoside equivalents (C3GE)/100 g DW). Fourteen phenolic compounds were identified in 50% acetone extract with quercetin derivatives and caffeoylquinic acids being the most abundant components [21]. Moreover, Zhang et al. [76] reported that 37 constituents, including 20 phenolic acids, 12 flavonoids, three organic acids, one ester, and one nucleoside, were identified in the ethyl acetate fraction of SPL extract, and 20 of them, such as caffeic acid ethyl ester, trans-N-feruloyltyramine, cis-N-feruloyltyramine, trans-N-(p-coumaroyl) tyramine, 4,5-feruloylcourmaoylquinic acid, indole-3- carboxaldehyde, 7,3′-dimethylquercetin, and 7-hydroxy-5-methoxycoumarin, were initially detected in SPL.

Flavonoid content of SPL also varies among sweet potato varieties, ranging from 18.0–72.7 QE mg/g [30]. Fu et al. [21] demonstrated that the TPC in SPL extracts ranged from 23.3–43.8 mg CAE/g DW, and 70% ethanol extract had the highest total anthocyanin (36.5 mg C3GE/100 g DW) and total flavonoid (3.4 mg QE/g DW) contents. The flavonoid compositions also vary greatly, depending on leaf color. Purple leaves of sweet potatoes contain cyanidin, quercetin, myricetin, and luteolin, while green leaves include apigenin [31,77]. Among flavonoid constituents, anthocyanins are the major compound occurring in substantial amounts in SPL [46,78,79], levels which are 2.5-fold higher than those in spinach [79]. The anthocyanin amount of SPL varies among sweet potato varieties. Ji et al. [80] found that purple SPL had much higher anthocyanin content than red-, yellow-, and green-colored SPL. SPL are also a source of carotenoids, and lutein is the major constituent occurring in SPL, ranging from 34–68 mg/100 g among sweet potato varieties [72]. Moreover, SPL also contain other phytochemicals, such as alkaloids, anthraquinones, oxalates, and steroids, at concentrations of 345.7, 328.4, 1.66, and 0.375 mg/100 g DW [21,70,81], respectively, whereas SPL contain lower amounts of phytic acid, cyanide, saponins, and tannins [81].

Since SPL are a significant dietary source of bioactive compounds, a comprehensive assessment of the compositions in leaves of sweet potatoes under various treatment methods (e.g., harvesting, cooking, drying, extraction methods) and cultivation conditions is warranted.

## 3. Antioxidant Activities

Various edible SPL are valuable sources of antioxidants in the diet. SPL marketed in different countries or areas widely vary in their antioxidant activities and may provide different health-promoting values. Leaves of sweet potato varieties with high antioxidant values can be processed for developing products with high nutraceutical values, providing good nutrition and improving human health. SPL contain various antioxidants [11,12,82], which contribute to the physiological defense against oxidative and free-radical-mediated reactions, leading to an increase in antioxidant defense and the suppression of low-density lipoprotein (LDL) oxidation and DNA damage in human lymphocytes [10,19,43]. Polyphenol antioxidants, especially caffeoylquinic acid derivatives (3,4,5-tri-O-caffeoylquinic acid, 3,5-di-O-caffeoylquinic acid, 3,4-di-O-caffeoylquinic acid, and 4,5-di-O-caffeoylquinic acid), exhibit strong antioxidant capacity [18,29,76,83]. In an in vivo study, Chang et al. [84] reported that the consumption of a high-polyphenol diet (200 g cooked purple SPL containing 5.7 mg GAE/g) for seven days modulated antioxidative status through dramatically enhancing the plasma total polyphenol level and ferric reducing ability of plasma, lowering the plasma IL-6 concentration, the thiobarbituric acid-reactive substance and protein carbonyl concentrations. Chang et al. [85] revealed that consumption of a purple SPL diet for two weeks modulated the antioxidant status of basketball players during the training periods through reducing lipid and DNA oxidation. In addition, purple SPL consumption influenced erythrocyte glutathione, plasma total antioxidant capacity, and plasma α-tocopherol [86,87]. Polyphenols occurring in SPL bring about an increase in glutathione by facilitating the expression of γ-glutamylcysteine synthetase [88] and inhibiting glutathione reductase [89].

SPL also can contain high levels of flavonoid antioxidants and flavonoids can significantly differ in their antioxidant capacity [90]. Green SPL are a rich source of quercetin, which was reported to exhibit three-fold more antioxidant capacity than eridictyol, and kaempferol [77]. Furthermore, anthocyanin was also considered as one of the most potent antioxidants of purple SPL [46]. Islam et al. [46] revealed the identification and characterization of 15 anthocyanins in SPL exhibiting both antioxidant and anti-mutagenic activities. In addition to anthocyanins, other phytochemicals, including triterpenes, alkaloids, saponins, anthraquinones, coumarins, and tannins also displayed the antioxidant activity in yellow SPL [21]. The antioxidant capacity of SPL was found to vary with the color of sweet potato leaves and to be higher than that of other leafy vegetables. Purple SPL exhibited higher antioxidant capacity than celosia (*Celosia argentea* L.), gynura (Gynura bicolor DC.), perilla (purple-leaved and bicolored-leaved) (*Perilla frutescens* (L.) Britton), edible amaranth (*Amaranthus tricolor* L.), heart leaf houttuynia (*Houttuynia cordata* Thumb.), and other commercial leafy vegetables, due to higher antioxidant content [22,77,91]. Ji et al. [80] found that the antioxidant capacity of purple SPL was significantly higher than other colored SPL (red, yellow, and white SPL). In a comparison of the oxidative capacity of six SPL varieties (‘Indon’, ‘Vitato’, ‘Oren’, ‘Biru-Putih’, ‘Batu-Biasa’, and ‘Batu-Kelantan’), ‘Biru-Putih’ and ‘Indon’ had the lowest and highest scavenging activities with IC_50_ of 597.61 µg/mL and 372.4 µg/mL, respectively [92]. In addition, Truong et al. [93] found a higher level of radical scavenging activity in leaves than in other plant parts in variety of sweet potato cultivars, including ‘Covington’, ‘Hernandez’, and ‘Beauregard’.

Therefore, SPL possess antioxidant properties that hold promise for applications of diet-mediated disease treatment and prevention. Further investigation focusing on the optimization of the SPL processing techniques (e.g., drying and extraction) in order to maintain the maximum content of antioxidant compounds is warranted.

## 4. Other Health Benefits

The search for dietary sources with potent biological activities has increasingly attracted considerable attention [94,95,96,97]. There is a great deal of interest in using potent dietary antioxidants in foods and pharmaceuticals to prevent oxidative reactions and chronic degenerative diseases [98,99,100]. SPL are a good source of nutrients, enhancing dietary protein, amino acid intake, and growth performance [66,67,101]. Furthermore, these major nutrients play a role in reducing the risks associated with certain diseases [5]. It was reported that SPL consumption can decrease the risks associated with cardiovascular disease due to the availability of complex carbohydrates, low-fat content, high dietary fiber [24,63]. Daily oral administration of purple SPL (200 g) can modulate various immune functions in human including elevated lytic activity of NK cells, secretion of cytokines IL-2 and IL-4, and increased proliferation responsiveness of peripheral blood mononuclear cells [19]. Since numerous health-promoting phytochemicals are found in SPL, regular intake of the leaves provides various health benefits. Among them, polyphenol constituents show various physiological functions and promote human health [24]. Leaves of sweet potato are rich in chlorogenic acid, a caffeoylquinic acid derivative, which is well-known for its health benefits, including protection against cancers [102], hypertensions [39], bacteria [69], diabetes [65], and heart disease [79]. Caffeoylquinic acids in sweet potato leaf is an angiotensin-converting enzyme inhibitor, controlling hypertension and congestive heart failure [39]. Among the health benefits of SPL, anti-cancer activity, hepato-protection, anti-inflammatory activity, antidiabetic activity, and antimicrobial activity are recognized as the major effects of SPL (Table 2). These benefits are therefore described as follows.

### 4.1. Anti-Cancer Activity

Leaves of sweet potato have been recognized as a potent anti-cancer food source against various cancer cells, including HCT-116 colon cancer [103], HeLa cancer [103], MCF-7 breast cancer [15], prostate cancer [16], colorectal cancer [104], and lung cancer [102] cells due to high content of anthocyanins [44,45], and polyphenols [7]. Methanol extracts of SPL inhibit proliferation of all human prostate cancer cells (PC-3, C4-2B, C4-2, DU145, and LNCaP) with IC_50_ values of 145–315 μg/mL due to modulations of cell cycle, inductions of apoptosis, and reductions of clonogenic survival [79]. The anti-prostate cancer activity of SPL is attributed to the presence of 3,4-di-O-caffeoylquinic acid, 3,5-di-O-caffeoylquinic acid, 4,5-di-O-caffeoylquinic acid, quinic acid, isochlorogenic acids, caffeic acid, and ester chlorogenic acid [16]. Chen et al. [42] showed that polyphenols in purple SPL depressed proliferation, migration, and tube formation of vascular endothelial growth factor-treated human umbilical vascular endothelial cells (HUVECs). Chlorogenic acid was reported as a strong and selective inhibitor of matrix metalloproteinase-2 [42] and matrix metalloproteinase-9 [105], which are angiogenic enzymes responsible for tumor metastasis and invasion, so that it can demonstrate several desirable anti-carcinogenic properties including inhibitory activity against A549 human lung cancer cells [102]. Caffeoylquinic acid derivatives also have potential for cancer prevention through apoptosis induction by increasing caspase-3 activity and expression of c-Jun (apoptosis-related gene) [7]. Among these compounds, 3,4,5-tri-O-caffeoylquinic acid effectively inhibits the development of human colon cancer DLD-1 cells, promyelocytic leukemia HL-60 cells, and stomach Kato III cancer cells, whereas caffeic acid demonstrates higher inhibitory activity against HL-60 cells than other di- and tri-O-caffeoylquinic acids [7]. Recently, a 16-amino-acid peptide, named the peptide *Ipomoea batatas* anti-cancer peptide (IbACP) from SPL, showed the inhibition of pancreatic cancer line [68]. Several studies have also been performed to evaluate the in vivo anti-cancer activity of SPL [6,16]. Gundala et al. [16] reported that the daily consumption of polyphenol-rich SPL extract (400 mg/kg) inhibited the growth and induced the apoptosis in both human prostate cancer cell and in vivo prostate cancer xenograft models. Similarly, the consumption of Okinawan SPL extract (200 ppm and 1000 ppm) for 12 weeks potentially inhibited the progression and development of neoplasms in mouse colon carcinogenesis model [6].

### 4.2. Hepato-Protection

The activity of anthocyanins of purple SPL was tested on carbon tetrachloride-treated human normal hepatocyte HL7702 cells [20], tert-butyl hydroperoxide-treated HepG2 cells, and rat hepatic stellate HSC-T6 cells [48]. The results demonstrate that anthocyanins of purple SPL (100–400 μg/mL) reduced the accumulation of reactive oxygen species (ROS) in TC-HL7702 cells [20], and inhibited the proliferation of HSC-T6 cells by inhibiting α-smooth muscle actin (SMA) expression, extracellular signal-regulated kinases 1 and 2 (ERK1/2), and the serine-threonine kinase Akt activation, and blocking platelet-derived growth factor receptor (PDGFR)-β signaling [48]. Furthermore, anthocyanins also reduced the cell death in t-BHP-treated HepG2 cells by lowering the levels of intracellular ROS, caspases-3 activity, lipid peroxidation, and by enhancing the levels of cytoprotective enzymes in HepG2 cells via ERK1/2/Nrf2 and Akt signaling pathways [48].

### 4.3. Anti-Inflammatory Activity

Extract of SPL and its constituents, cyanidin and quercetin, were observed to show anti-inflammatory effects via reducing the mitogen-activated protein kinase (MAPK), ERK1/2 expression, and nuclear factor kappa B (NFκB), inhibiting tumor necrosis factor-α (TNF-α)-induced monocyte-endothelial cell adhesion, and attenuating interleukin-8 (IL-8), vascular cell adhesion molecule-1 (VCAM-1), and CD40 (a member of TNF receptor family of cell surface proteins) expression [50]. The consumption of purple SPL can modulate various immune functions, including secretion of cytokines IL-2 and IL-4 of NK cells and can induce an increase in proliferation responsiveness of peripheral blood mononuclear cells due to the high polyphenol content of the leaves [19]. Moreover, the purple SPL extracts depress neuroinflammatory responses in lipopolysaccharide-activated BV-2 microglia cells by inhibiting production of pro-inflammatory mediators, such as inducible nitric oxide synthase (iNOS), cyclooxygenase 2 (COX-2), nitric oxide (NO), and TNF-α. The anti-neuroinflammatory potential of SPL extract was considered to be related to its strong antioxidant properties [106].

### 4.4. Antidiabetic Activity

SPL contain several constituents that show a potential activity against diabetes. Chlorogenic acid reduces the release of glucose into the blood-stream, lowering the glycemic index, thereby benefitting diabetic patients and reducing the risk of type II diabetes [41]. In addition, the polyphenol contents in leaves of 116 sweet potato cultivars grown in China showed anti-diabetic activity [38]. In an in vivo study, the extract of SPL can reduce the blood glucose levels of both STZ-induced diabetic and healthy rats, indicating hypoglycemic and anti-hyperglycemic activities of SPL by stimulating glucagon-like peptide-1 (GLP-1) secretion [48]. The maximum hypoglycemic activity of the extracts in both STZ-induced hyperglycemic and healthy rats were obtained at the dose of 400 mg/kg with non-cytotoxicity [48]. In another in vivo study, the consumption of SPL (3% in diet) for 5 weeks modulated the hypoglycemic activity in type-2 diabetic mice [47]. Phenolic acids, caffeoylquinic acid derivatives, and anthocyanins were found to be one of the key hypoglycemic contributors in SPL [47]. In addition, Zhang et al. [38] reported that phenethylcinnamides and 3,4,5-tri-O-caffeoylquinic acid from SPL manifested strong α-glucosidase inhibition. Lin et al. [107] described that purple, yellow, and red SPL extracts in 70% ethanol considerably promoted expressions of glucose transporter (GLUT)-2 relative to that of a tumor necrosis factor-α-treated group in insulin-resistant FL83B hepatocyte cells, and lowered risk of diseases such as type II diabetes.

### 4.5. Antimicrobial Activity

SPL also exhibit potential for antimicrobial activity. Islam [69] reported the potent antibacterial activity of the leaf extract of three sweet potato cultivars against *Staphylococcus aureus*, *Bacillus cereus*, and *E. coli* O157:H7. Polysaccharides are considered to be the major anti-bacterial agents in SPL extract [69]. However, ethanol leaf extract of Brazilian sweet potato did not show any anti-bacterial and anti-fungal activities against *S. aureus*, *S. mutans*, *S. mitis*, and *Candida albicans* [70]. This result could be attributed to differences in the methodology of antimicrobial tests and phytochemical compositions in the SPL [70]. There are still limited studies on the antimicrobial activity of SPL, thus, further studies are needed to clarify SPL antimicrobial activity and the mechanisms involved.

SPL have shown potential applications in provision of human health benefits, including the reduction of oxidative damage and the prevention of some diseases. Those health-promoting benefits are attributed to the presence of various constituents having strong bioactivity. Therefore, SPL can be an alternative natural dietary source providing additional applications in the food supplement and nutraceutical industries.

## 5. Relationships between *In Vivo* and *In Vitro* Studies

SPL have been widely consumed for human diet and many studies have been conducted to examine their health-benefit effects [107,108]. However, very few studies have been performed to compare the in vitro and in vivo activity of SPL. Karna et al. [79] reported the in vitro and in vivo anticancer activity of SPL against prostate cancer. The polyphenol-rich SPL extract showed significant antiproliferative activity by modulating cell cycle and apoptosis regulatory molecules, reducing clonogenic survival, perturbing cell cycle progression, and inducing apoptosis in human prostate cancer PC-3 cells (in vitro) and prostate tumor xenografts model (in vivo) [79]. In another study, SPL suppressed oxidation of LDL in vitro and in vivo [10]. SPL exhibited a radical scavenging effect and prolonged LDL oxidation lag time in vitro. In a clinical trial, healthy volunteers (7 female and 6 male) consuming SPL (18 g) also demonstrated a decrease in LDL mobility and prolonged LDL lag time [10]. There was a correlation between total TPC and antioxidant activity of SPL [10]. Chen et al. [42] investigated the inhibitory influence of SPL on angiogenesis in human umbilical vascular endothelial cells (in vitro) and in human serum (ex vivo). There was no correlation between in vitro and ex vivo results [42]. The methanol extract of SPL were anti-angiogenesis in vitro, but the ex vivo study demonstrated pro-angiogenetic. This could be because the differences in the chemical compositions between leave metabolites in human serum and leaf extract, thus causing the opposite effect [42]. Generally, no studies have established mathematical models to describe correlation between in vitro and in vivo results. Therefore, further studies are required to examine that relationship.

## 6. Conclusions and Perspectives

Leaves of various sweet potato varieties (red, yellow, purple, green, white flesh) have their unique bioactive compositions, and polyphenols and flavonoids are considered as major constituents. Due to high content of such bioactive compounds, many great potential health-promoting benefits, including anti-oxidation, anti-diabetics, anti-cancer, anti-hepatotoxicity, anti-inflammation, and anti-bacteria have been observed in these SPL. However, more human health studies and clinical trials should be performed to validate the health-promoting benefits of various sweet potato leaves. In addition, identification of the complete profiles of phytochemicals of sweet potato varieties in relation to their bio-activity is also needed. Generally, SPL can also serve as a promising natural dietary resource and can be further developed as a sustainable crop for use in the food and medicinal industries.

## Figures and Tables

**Table 1:** Mineral and vitamin compositions of sweet potato leaves.

Elements	Quantity (mg/100 g DW)	References
Na	8.06−832.31	[5]
Mg	220.2−910.5	[5]
P	131.1−2639.8	[5]
Ca	229.7−1958.1	[5]
K	479.3−4280.6	[5]
Cu	0.7−1.9	[5]
Zn	1.2−3.2	[5]
Mn	1.7−10.9	[5]
Fe	1.9−21.8	[5]
Niacine (vitamin B3)	0.856−1.498	[2]
Vitamin B6	0.12−0.329	[2]
Vitamin B2	0.248−0.254	[2]
Vitamin B1	0.053−0.128	[2]
Vitamin C	0.0627−0.081	[2]
Vitamin E	0.00139−0.00284	[2]
Pantothenic acid (Vitamin B5)	0.32−0.66	[2]
β-carotene	0.273−0.4	[2]
Biotin	0.003−0.008	[2]

**Table 2:** Health benefits of bioactive compounds in sweet potato leaves.

Compounds		Function	References
Phenolic acids	Caffeic acid derivatives	Antioxidant activity	[29,32]
		Anti-mutagenic	[18]
		Antidiabetic	[32]
		Anticancer	[16,22]
		Anti-inflammatory	[33,34,35]
	Caffeoylquinic acid derivatives	Antioxidant activity	[29,36,37,38]
		Anticancer	[7,15,16,17]
		Anti-hypertension	[39]
		Antidiabetic	[38]
		Heart protection	[15,40]
	Chlorogenic acid	Antidiabetic	[41]
		Anticancer	[15,16,42]
	Quinic acid	Anticancer	[15,16]
Flavonoids	Anthocyanins	Antioxidant activity	[43,44,45]
		Anti-mutagenic activity	[46]
		Anticancer	[44,45]
		Hypoglycemic activity	[47]
		Hepato-protection	[20,48]
		Anti-inflammatory	[49,50]
	Quercetin	Antioxidant activity	[28,43]
		Anticancer	[15]
		Anti-inflammatory	[50]
	Apigenin	Anticancer	[51]
	Kaempferol	Anticancer	[15]
	Myricetin	Anticancer	[52]
		Antidiabetic	[52]
	Fisetin	Anticancer	[53]
		Anti-inflammatory	[54]
	Morin	Anticancer	[55]
		Anti-inflammatory	[56]
	Isorhamnetin	Cardioprotection	[57]
	Luteolin	Anticancer	[58]
		Anti-inflammatory	[59]
Mono-and di-galactosyldiacylglycerol		Anti-inflammatory	[60]
Carotenoids		Anti-cancer	[61]
		Cardioprotection	[61]
Dietary fiber		Antioxidant activity	[62]
		Cardioprotection	[63]
		Anticancer	[64]
		Antidiabetic	[65]
Dietary protein		Growth performance enhancement	[66,67]
16-amino acid-peptide (IbAcp)		Anticancer	[68]
Polysaccharides		Antibacterial activity	[69]
		Antifungal activity	[69,70]
Omega-3 fatty acids		Cardioprotection	[71]
		Anti-inflammatory	[71]
Alkaloids		Antioxidant activity	[21]
Saponins		Antioxidant activity	[21]
Coumarins		Antioxidant activity	[21]
Tannins		Antioxidant activity	[21]
